# Complete Genome Sequence of a Novel Swine Acute Diarrhea Syndrome Coronavirus, CH/FJWT/2018, Isolated in Fujian, China, in 2018

**DOI:** 10.1128/MRA.01259-18

**Published:** 2018-12-06

**Authors:** Kai Li, Hao Li, Zhen Bi, Jun Gu, Wang Gong, Suxian Luo, Fanfan Zhang, Deping Song, Yu Ye, Yuxin Tang

**Affiliations:** a Key Laboratory for Animal Health of Jiangxi Province, Nanchang, Jiangxi, China; b Department of Preventive Veterinary Medicine, College of Animal Science and Technology, Jiangxi Agricultural University, Nanchang, Jiangxi, China; KU Leuven

## Abstract

The full-length genome sequence of a novel swine acute diarrhea syndrome coronavirus (SADS-CoV), CH/FJWT/2018, was determined, which was genetically most closely related to CN/GDWT/2017, recently discovered in Fujian, China. The indel sites of the spike (S) gene of CH/FJWT/2018 were most similar to those of bat-origin SADS-related coronaviruses.

## ANNOUNCEMENT

Swine acute diarrhea syndrome coronavirus (SADS-CoV) belongs to the genus Alphacoronavirus in the family Coronaviridae and is an enveloped, positive-sense, single-stranded RNA virus (1). In January 2017, SADS-CoV was first discovered in suckling piglets with severe enteritis in South China after ruling out the presence of porcine epidemic diarrhea virus (PEDV), transmissible gastroenteritis virus (TGEV), porcine deltacoronavirus (PDCoV), and other known pathogens associated with piglet diarrhea (2–8). SADS-CoV had 95% nucleotide (nt) identity at the full-genome level with the previously reported bat-HKU2 strains (3, 4).

In this study, fecal and small intestinal samples (*n* = 168) collected from diarrheal piglets from seven farms in Fujian Province, China, in 2018 were tested for the presence of SADS-CoV based on reverse transcription-PCR (RT-PCR), as described previously (6). A SADS-CoV, designated CH/FJWT/2018, was identified, and this virus was isolated in Vero cell cultures according to the established protocol in the Key Laboratory for Animal Health of Jiangxi Province. To determine the complete genome sequence of CH/FJWT/2018, total RNA was extracted with TRIzol reagent (Invitrogen, USA) according to the manufacturer’s instructions, and 36 sets of primers covering the complete genome of CH/FJWT/2018 were synthesized and then employed for RT-PCR. Each amplicon, including the 5′ and 3′ ends amplified by a Clontech SMARTer rapid amplification of cDNA ends (RACE) 5′/3′ kit (TaKaRa, Japan), was sequenced with the Sanger sequencing method (Sangon Biotech, Ltd., China). The full-length genome sequence was then assembled and analyzed by Lasergene v7.10 (DNAStar, Inc., USA).

The complete genome sequence of CH/FJWT/2018 determined was 27,169 nt in length, excluding the 3′ poly(A) tail. The genomic arrangement and corresponding nucleotide positions were as follows: open reading frame 1a (ORF1a), nucleotides 304 to 12456; ORF1b, nucleotides 12456 to 20489; spike gene, nucleotides 20486 to 23881; ORF3, nucleotides 23881 to 24570; envelope gene, nucleotides 24551 to 24778; membrane gene, nucleotides 24787 to 25476; and nucleocapsid gene, nucleotides 25488 to 26615. The results of a phylogenetic analysis using Molecular Evolutionary Genetics Analysis (MEGA) v7.0 (9), based on CH/FJWT/2018 and other reference sequences of SADS-CoVs retrieved from GenBank, showed that CH/FJWT/2018 had 99.6% nt identity with CN/GDWT/2017 (GenBank accession number MG557844), a swine-origin SADS-CoV, and 98.9% nt identity with bat CoV HKU2 (EF203064) (10, 11), respectively. The complete genome sequence of CH/FJWT/2018 is 3 nt longer than that of HKU2. The mutations that occurred included a 3-nt (TTG) insertion between nucleotides 4554 and 4555 in the ORF1a gene, a 6-nt (GGCCTC) insertion between nucleotides 20504 and 20505, a 3-nt (GGC) deletion between nucleotides 22463 and 22465 in the spike (S) gene, and a 3-nt (GTA) deletion between nt 24773 and 24775 in the membrane (M) gene. Interestingly, the insertion/deletion patterns of CH/FJWT/2018 were more similar to those of bat-origin SADS-related coronaviruses (SADSr-CoVs) (6) than to those of swine-origin SADS-CoVs, suggesting that CH/FJWT/2018 might originate from bats. The findings determined in this study suggest that CH/FJWT/2018 was a novel SADS-CoV, and further study is required to address its biological properties, etiologies, and pathogenesis.

### Data availability.

The CH/FJWT/2018 sequence data have been deposited in GenBank under the accession number MH615810.
